# Fantasy Proneness Correlates With the Intensity of Near-Death Experience

**DOI:** 10.3389/fpsyt.2018.00190

**Published:** 2018-06-07

**Authors:** Charlotte Martial, Héléna Cassol, Vanessa Charland-Verville, Harald Merckelbach, Steven Laureys

**Affiliations:** ^1^Coma Science Group, GIGA-Consciousness and Neurology Department, University and University Hospital of Liège, Liège, Belgium; ^2^Forensic Psychology Section, Maastricht University, Maastricht, Netherlands

**Keywords:** fantasy proneness, near-death experience, experiencer, near-death experience-like, creativity

## Abstract

Little is known about the personality characteristics of those who have experienced a “Near-Death Experience” (NDE). One interesting candidate is fantasy proneness. We studied this trait in individuals who developed NDEs in the presence (i.e., classical NDEs) or absence (i.e., NDEs-like) of a life-threatening situation. We surveyed a total of 228 individuals. From those, 108 qualified as NDE experiencers (i.e., Greyson NDE scale total score ≥7): 51 had their NDEs in the context of a life-threatening situation; 57 had their NDEs not related to a life-threatening situation. From those who did not meet the criteria to be considered “experiencers,” 20 had their NDE in the absence of a life-threatening situation; 50 had faced death but did not recall a NDE and finally, 50 were healthy people without a history of life threat and/or NDE. All participants completed a measure of NDE intensity (the Greyson NDE scale) and a measure of fantasy proneness (the Creative Experiences Questionnaire). People reporting NDEs-like scored higher on fantasy proneness than those reporting classical NDEs, individuals whose experiences did not meet the NDE criteria and matched controls. By contrast, individuals reporting classical NDEs did not show different engagement in fantasy as matched controls. The reported intensity of the experiences was positively correlated with engagement in fantasy. Our findings support the view that strong engagement in fantasy by individuals recalling NDEs-like might make these persons more likely to report such subjective experiences when exposed to suitable physiological and/or psychological conditions (e.g., meditation, syncope).

## Introduction

When facing a life-threatening situation—or, at least, a situation perceived as such, some people will later report having lived various phenomenological experiences (e.g., seeing a bright light and/or a dark tunnel, out-of-body experiences, encountering deceased relatives) that are intriguing by their paranormal appearance or are surprising by their extra-ordinary aspect (1). These distinct perceptual experiences are commonly referred to as “Near-Death Experiences” (NDEs). To explain NDEs and their phenomenal content, three main non-mutually exclusive explanatory models have been proposed (2, 3). Spiritual theories assume a “dualistic” approach toward the mind-brain relationship (4), neurobiological approaches suggest that specific brain networks and functions might underlie NDEs (5, 6), whereas psychological theories have advocated that NDEs are the result of a dissociative defense mechanism in response to extreme danger (7). To facilitate the identification of NDEs and to assess core content components and their intensity, researchers have used the Greyson NDE scale (the Greyson NDE scale; (8)]. Yet, looking at the empirical evidence [see (9) for review], the possible causes and conditions under which NDEs appear remain unclear.

Intriguingly, similar phenomenological experiences termed “NDEs-like” (i.e., containing a comparable phenomenological content and intensity) have been reported in situations where there was no genuine threat to the individuals' life (10, 11), such as during a meditative state (12) or intense grief and anxiety (13). Gabbard and Twemlow (14) argued that the expectancy of an impending death or the strong belief of one's death felt at that moment, rather than the actual proximity of death, would suffice to trigger NDEs. However, given that some NDEs-like do not include any perceived threat to life, not all experiences in this category can be explained by the expectancy or the belief in an impending death (11). Although enigmatic, the NDE-like phenomenon has been the subject of very few empirical studies (10, 11, 14).

The current paper focuses on fantasy proneness (15) as one potential variable that may shape people's reports of NDEs and NDEs-like. Fantasy proneness refers to a habitual engagement in imaginative activities (16). Each individual has (to some extent) imaginative capacities and can report a range of experiences that are more or less related to imagination [e.g., daydreaming; (17)]. Yet, even within non-psychiatric samples, fantasy can distort perception and memory thereby leading to reality monitoring errors (17).

Cognitive researchers have argued that we constantly use information gathered through the senses (“bottom-up” processing) but we also construct meaning about our environment (and our interactions with it) by using information we have already stored in memory (“top-down” processing). Indeed, our brain is constantly trying to make sense of the world around us and the information it receives by using this general dynamic process of two-way flow of information (18). In the case of NDEs, the phenomenal content would reflect individuals' attempts to make sense of the ambiguous perceptive feature of NDEs in circumstances (i.e., during altered state of consciousness) that enhance such ambiguous bottom-up information (9, 19). Related to this perspective are theories that emphasize depersonalization and other dissociative symptoms. When facing a life-threatening or aversive situation, a person may feel disconnected from the external world and focus attention on internally oriented fantasies (7, 15, 20). Yet, this account assumes that the eliciting event was threatening and it is not clear how it would explain NDEs-like phenomena.

Although many humans are exposed to life-threatening situations (i.e., a severe brain injury) or have the feeling that they have been close to death at some point in their life, only a limited number of persons recall identifiable “classical” NDEs [i.e., (21, 22)]. Similarly, an important question is why some individuals experience NDEs-like phenomena, when others do not, although they have been exposed to physiological and/or psychological conditions that are known to be associated with NDEs-like (e.g., meditation, syncope). People reporting “classical” NDEs (i.e., with a context of a life-threatening situation) do not seem to show any deficits in global cognitive functioning (21), but Martial et al. (23) observed less optimal source monitoring abilities and heightened illusory recollections in this population. In addition, Greyson (24) found that experiencers report more dissociative symptoms than people who had come close to death without subsequently developing NDEs. As fantasy proneness is strongly related to dissociative symptoms and source monitoring breakdown (25), these findings suggest that people with NDEs score higher on fantasy proneness than those without NDEs. An unpublished conference presentation found stronger imaginative activities [as indexed by the Inventory of Childhood Memories and Imaginings; (26)] in experiencers reporting classical NDEs compared with those reporting an event that brought them near to death but did not feel that they had a NDE or with healthy subjects (Council and Greyson, unpublished data, 1985). By contrast, using the Childhood Experience Inventory, Ring and Rosing (27) did not find more pronounced imaginative involvement in people reporting NDEs. These conflicting findings may be due to the different instruments that the authors employed to measure fantasy proneness.

With this in mind, the present study aimed to assess fantasy engagement in experiencers reporting NDEs [i.e., the memory scored 7 or above on the Greyson NDE scale; (8)] (1) in and (2) outside the context of an actual threat to life (i.e., NDEs-like); (3) in individuals reported having had a NDE without a life-threatening situation but not qualified as experiencers [i.e., the memory scored below the cutoff score of 7 on [the Greyson NDE scale; (8)]; (4) in matched control participants who had been exposed to a life-threatening situation but did not report any kind of NDE; and finally (5) matched control participants who had neither faced a life threatening situation, nor had any NDEs. To this end, the Creative Experiences Questionnaire [CEQ; (16)] was administered to all groups. We also looked into the association between fantasy proneness (i.e., CEQ total score) and self-reported intensity of NDEs [i.e., Greyson NDE scale total score; (8)].

## Materials and methods

### Participants

Subjects were recruited from among individuals who contacted us to share their experience. Initially, they were recruited via the International Associations for Near-Death Studies (IANDS France and Flanders) and the Coma Science Group (GIGA-Consciousness, University and University Hospital of Liège, Belgium). Control participants were recruited via announcements by the Coma Science Group. The study was approved by the ethics committee of the Faculty of Medicine of the University of Liège.

The total sample consisted of 128 people who claimed to have experienced a NDE. Reported experiences were assessed according to the Greyson NDE scale (8). Fifty-one participants (40%) described experiences that met the accepted criteria of NDEs [i.e., Greyson NDE scale total score ≥7/32; (8)] in the context of a life-threatening situation (“NDErs/LTS” group). Fifty-seven (44%) described experiences that also met the accepted criteria of NDEs [i.e., Greyson NDE scale total score ≥7/32; (8)] but in the absence of a life-threatening situation (“NDErs/non-LTS” group). Twenty (16%) described experiences in the absence of a life-threatening context but that did not meet the accepted criteria of NDEs (i.e., Greyson NDE scale total score <7/32; (8); “non-NDErs/non-LTS” group). We recruited a cohort of 100 control participants: 50 people who had come close to death at some point in their lives but did not recall a NDE (“controls/LTS” group) and 50 healthy people who had never reported to have experienced NDEs (“controls/non-LTS” group). Completion of the anonymous questionnaire was voluntary and written informed consent was obtained from all participants enrolled in the study.

### Measures

Participants were invited to participate in a study on creativity. To that end, they were mailed a questionnaire including the Creative Experiences Questionnaire [CEQ; (16)] and items of socio-demographic (gender, age) and clinical (time since experiences, presence of life-threatening event) data. To gauge the presence of a life-threatening event (i.e., a severe brain injury), we asked participants whether they had gone through a period of coma and whether they had stayed in intensive care. The term “fantasy proneness” was not used either in the explanatory letter or in the questionnaire itself. The CEQ (16) is a self-report instrument which is a measure of fantasy proneness including 25 true/false items. A total score is derived from the sum of all the true responses and referred to as a validated index of propensity toward fantasy [higher scores indicate higher levels of fantasy proneness; (16)]. Illustrative items are: “As a child, I had my own make believe friend or animal,” “Many of my fantasies have a realistic intensity,” and “When I imagine I have eaten rotten food, I really get nauseous.”

The Greyson NDE scale is a validated 16-item multiple-choice tool used to obtain a standardized identification of NDEs with a validated cut-off score of 7 (8). For each item, scores are arranged on an ordinal scale ranging from 0 to 2. This scale also permits the intensity of the NDE to be quantified (i.e., total score ranging from 0 to 32), because it considers both the number of experienced dimensions (i.e., item marked 0 = “not present” or present) and the gradation of intensity in the scoring provided (i.e., 1 = “mildly or ambiguously present” and 2 = “definitively present”). The instrument includes four subscales: cognitive, affective, paranormal, and transcendental components. Participants whose experience did not meet the accepted criteria [i.e., total score <7/32; (8)] were included in the “non-NDErs/non-LTS” group (see below).

### Analyses

Pearson's χ^2^ tests were used to assess frequency distributions. One-way ANOVAs and *t*-tests were performed to compare age, age at experience, time since experience, and reported intensity of the NDE within groups. Pairwise planned comparisons were then conducted to determine which groups differed significantly from each other. The distribution of CEQ total scores was skewed. For this reason, non-parametric tests were used. Thus, group differences regarding the CEQ were evaluated with the Kruskal–Wallis test. Next, we performed *post-hoc* comparisons using Bonferroni-corrected (*p* < 0.01) Mann–Whitney *U*-tests to examine possible differences across groups. Spearman's rank-order correlations were computed to examine associative strength between CEQ total scores and Greyson NDE scale total scores and subscale scores. All participants who reported having experienced a NDE were included, also those who obtained a score of <7 on the Greyson NDE scale. In addition, we calculated Spearman rank-order correlations between CEQ and Greyson NDE total scores for each of the two experiencer groups separately. Finally, we used Fisher's *r*-to-*z* transformation to test the significance of the difference between these two correlation coefficients. To avoid type I errors, Bonferroni adjustments (*p* < 0.007) were applied.

## Results

The five groups did not significantly differ with regard to gender and age distributions (see Table 1). Experiencers groups and the non-NDErs group did not differ either for age at experience or for time elapsed since the experience.

**Table 1:** Demographic data of subsamples.

**Demographics**	**NDErs**		**Non-NDErs non-LTS (*n* = 20)**	**Controls**		***p***	**η^2^**
	**LTS (*n* = 51)**	**non-LTS (*n* = 57)**		**LTS (*n* = 50)**	**non-LTS (*n* = 50)**		
Gender–female	27 (53%)	36 (63%)	11 (55%)	32 (64%)	29 (58%)	0.76	–
Age	57 ± 13	57 ± 14	62 ± 14	53 ± 11	55 ± 12	0.11	0.03
Mean in years ± *SD*							
Age at experience	35 ± 17	28 ± 16	35 ± 20	29 ± 13	–	0.14	0.03
Mean in years ± *SD*								
Time since experience	22 ± 16	28 ± 17	27 ± 20	24 ± 12	–	0.16	0.03
Mean in years ± *SD*							

*NDErs, near-death experiencers; LTS, life-threatening situation; SD, standard deviation*.

As to the intensity (i.e., Greyson NDE scale total score) of reported NDEs, NDErs/LTS (mean total score = 16 ± 5), and NDErs/non-LTS (mean total score = 15 ± 6) groups did not score differently [*t*_(106)_ = 0.25, *d* = 0.20, *p* = 0.79].

Total CEQ scores were significantly different between groups (see Table 2; see Supplementary Material for endorsement percentages for each CEQ item across subsamples). The NDErs/non-LTS group obtained significantly higher CEQ total scores than the NDErs/LTS (*p* < 0.005, *d* = 0.56), non-NDErs/non-LTS (*p* < 0.001, *d* = 0.83), and control/non-LTS (*p* < 0.0001, *d* = 1.2) groups. By contrast, CEQ total scores of NDErs/LTS and controls/LTS groups were not significantly different (*p* = 0.019, *d* = 0.47). Finally, non-NDErs/non-LTS and control/non-LTS groups did not obtain different CEQ total scores (*p* = 0.39, *d* = 0.21).

**Table 2:** CEQ total scores of subsamples (on the first line: total scores summing all items; on the second line: total scores excluding the three items overlapping with the Greyson NDE scale items).

**Scale**	**NDErs**		**Non-NDErs non-LTS (*n* = 20)**	**Controls**		***p***	***d***
	**LTS (*n* = 51)**	**non-LTS (*n* = 57)**		**LTS (*n* = 50)**	**non-LTS (*n* = 50)**		
CEQ total score	7 (5-11)	11 (7-13)	7 (4-8)	6 (3-9)	6 (2.5–8)	<0.0001	0.85
Median (IQR)							
CEQ total score (excluding item 21, 23, and 25)	7 (4–9.5)	9 (5-12)	6 (4-7)	5 (3-8)	6 (2-8)	–	–
Median (IQR)							

*NDErs, near-death experiencers; LTS, life-threatening situation; IQR, inter-quartile range*.

Among individuals who claimed to have had a NDE (whether or not they reached the cut-off of 7/32 on the Greyson NDE scale; i.e., NDErs/LTS, NDErs/non-LTS, and non-NDErs/non-LTS groups), Greyson NDE scale total scores were positively correlated with total CEQ scores (see Table 3). To investigate whether the correlational result observed in this analysis effectively reflects an association between experiencers' investment in fantasy and the reported intensity of the NDE, we performed a Spearman's rank-order correlation between Greyson NDE scale and CEQ total scores but without including the three CEQ items (item 21, 23, and 25) showing some overlaps with certain items of the Greyson NDE scale. We observed a similar significant positive correlation (Spearman *r* = 0.26; *p* < 0.005; see Table 2 for raw median CEQ scores without the three items).

**Table 3:** Spearman rank correlations between CEQ total score and Greyson NDE scale scores and subscale scores for the total sample (*n* = 128).

	**CEQ total score**	***p***
**GREYSON NDE SCALE**		
Total score	0.32	<0.0005
**SUBSCALE SCORES**		
Cognitive	0.22	0.015
Affective	0.27	<0.007
Paranormal	0.26	<0.007
Transcendental	0.28	<0.007

Total CEQ scores were also positively correlated with affective, paranormal and transcendental subscale scores but not with cognitive subscale scores (Table 3). Looking at the subsamples, for the NDErs/non-LTS group only, we found a significant positive correlation (Spearman *r* = 0.33; *p* < 0.007) between Greyson NDE scale total scores and total CEQ scores. By contrast, in the NDErs/LTS group, the correlation between both total scores did not attain significance (Spearman *r* = 0.28; *p* = 0.054). Nevertheless, the Fisher's *z* test showed that the two associations were not significantly different (*z* = 0.28; *p* = 0.39).

## Discussion

The sample of individuals who reported NDEs-like scored higher on self-reported measures of fantasy proneness than matched control individuals who had never experienced NDEs and individuals who had reported similar experiences that did not meet NDEs criteria. Interestingly, although both groups of experiencers did not differ in terms of intensities of experience [as also reported in previous studies; (10)], experiencers recalling NDEs-like showed a greater engagement in fantasy than those with classical NDEs. Thus, compared with control participants who exhibited moderate fantasy engagement [in line with previous non-clinical population studies; (16, 28)], the CEQ scores of individuals who reported NDEs-like is suggestive of heightened fantasy proneness levels.

The retrospective and correlational design of this study does not permit conclusions to be made about the casual pathway; that is, whether NDEs-like occur more frequently in individuals with (previously established) high engagement in fantasy or whether such experiences encourage fantasy proneness in individuals who were previously not prone to fantasy. Yet it is reasonable to hypothesize that high engagement in fantasy, as a habitual tendency, makes people more likely to report subjective NDEs-like when exposed to certain physiological and/or psychological conditions (e.g., meditation, syncope). Indeed, some items of the CEQ allude to retrospective recall of childhood experiences (16), supporting the idea of an enduring predisposition toward fantasy in those who score relatively high on the CEQ. It can then be assumed that the core experience of a NDE would be common to all experiencers and physiologically determined. However, top-down mechanisms would then interact by influencing mnesic details of the content and the interpretation thereof. Any unusual sensations—potentially engendered by a disrupted brain—or “fantasy guesses” (29) could then be integrated into the individual's model of reality, which could account for some elements perceived during NDEs.

By contrast, we found no indication that individuals with classical NDEs are more fantasy prone than matched controls, including individuals who had come close to death without having NDEs. Given the sample size in the current study, we believe that there is little reason to believe that a NDE *per se* is the result of fantasy prone fabrication.

Interestingly, we found an association between experiencers' investment in fantasy and imagination and the reported intensity of the NDE. More specifically, individuals' engagement in fantasy were correlated with affective, paranormal, and transcendental NDE features [as assessed by the Greyson NDE subscale scores; (8)], but not with their cognitive features. Yet, the correlational analyses performed within each of the two experiencers' groups revealed differential patterns depending on whether (or not) a threat to the individual's life was experienced. That is, the more individuals described intense NDEs in the absence of a life-threatening situation, the higher they scored on fantasy proneness, whereas there was no significant correlation between the intensity of the NDE and an engagement in fantasy for experiencers reporting classical NDEs. Nevertheless, when comparing the two correlation coefficients obtained in the two experiencers' groups, we did not observe difference in the strength of association between the engagement in fantasy and the intensity of the NDE. Again, these correlations (or their absence) do not imply any form of causality (or the absence thereof). Still, another hypothesis is that when no life-threatening situation was present, the reported intensity of the experience depends on how strongly the individual is involved in fantasy and imaginative processes. In the extant literature, functional neuroimaging studies have demonstrated that spontaneous thoughts [accompanied by a disengagement from the external environment; (30)] are constrained by the organization of our neurocognitive system even though they arise independently of external input (31). While it appears that subjective NDEs are intensively experienced by the experiencers' inner world and that variation in spontaneous thoughts is (at least partly) constrained by the network organization of the brain (31), further studies should investigate structural and functional connectivity profiles using neuroimaging techniques in people who have recalled NDEs and people who do not. Moreover, whether classical NDEs and NDEs-like phenomena are underpinned by similar neural mechanisms remains an unanswered question.

The present findings warrant follow-up investigation. Specifically, it is important to look into factors (e.g., reality monitoring failures) involved in fantasy proneness that may generate NDEs-like. One possibility might be that individuals with NDEs-like are more “open to experiences,” a personality trait of the Five-Factor Model (32). It is likely that the experiencers recalling NDEs-like are unusually sensitive to internal states and possess a special propensity to pick up certain perceptual elements that other individuals are blind to. This formulation is consistent with the notion of a “NDE-prone personality,” defined as “the capacity to shift into states of consciousness that afford access to non-ordinary realities coupled with strong tendencies toward psychological absorption” (27). In line with this, fantasy prone individuals' lives appear to be experientially richer (33) and as a consequence, their fantasies are “as real as real” (15). This is consistent with Thonnard et al.'s (34) results showing that experiencers usually report a sense of “hyper-reality” regarding their experiences (due to extremely vivid memories).

Alternatively, the reality monitoring model (35) could provide a framework for understanding why some individuals may report NDEs-like accounts. The CEQ (16) used in this study specifically assesses the frequency in which individuals engage in fantasy and their difficulties in distinguishing fantasy and reality. Thus, it may be the case that these experiences arise as a consequence of source-monitoring errors, whereby inner thoughts and feelings are wrongly interpreted as memories of events that occurred in reality. Fantasy proneness goes along with lenient criteria and is closely related to suboptimal reality monitoring (35, 36). Thus, extreme internal focus in individuals could, in some cases, result in memories of subjective experiences meeting the identification criteria of NDEs but occurring without a life-threatening situation (i.e., NDEs-like).

In addition to potential influences of top-down mechanisms in the emergence of NDEs-like, it would be interesting to investigate top-down influences in the meaning, form, and content of the experience itself. In keeping with the reconstructive view described above, the NDE experiencers' religiosity and cultural background have been suggested to influence the NDEs' content and the features (29, 37, 38). However, most of the conducted studies are case studies and surveys are mainly conducted in Western cultures, thus limiting the generalizability and the conclusions that can be drawn. Translated versions of the Greyson NDE scale are now available [e.g., see for the Italian version: (39)], thereby facilitating future cross-cultural studies.

In line with the present findings and as previously stressed (11), NDEs-like phenomena call for a reappraisal of the (more general) NDE phenomenon. The label itself—NDE—does not appear to adequately describe the diversity of experiences (3, 6). Because there is no clear universal definition of NDEs, an implicit consensus between investigators has emerged where NDEs were defined in terms of their commonalities (40). However, while similar phenomenal content has been described for various states of consciousness [e.g., trance states, general anesthesia; (10, 11)], it appears that the classic features of the NDE phenomenon are not associated exclusively with actual confrontation with life-threatening circumstances. We believe that NDE research might benefit from employing a more fine-grained classification. Finally, we think that it is also important for NDE experiencers themselves. Indeed, the NDE label *per se* might contribute to the reluctance of people with NDEs-like phenomena to talk openly about their experience: some of them might consider their experiences as deviant because they were not in a critical context of impending death.

Our study has several methodological constraints. First, participants enrolled in the study were mostly self-selected and might not be representative due to a possible selection bias. Importantly, the personality construct of fantasy proneness itself could have biased the recruitment. Indeed, fantasy prone individuals might be less reluctant to share their experience as they could be more accustomed to dealing with non-ordinary experiences. Second, our study was cross-sectional and relied on self-report measures. Third, it should be point out the lack of medical information regarding the presence of a life-threatening event. Finally, one can ask to what extent there is an overlap between the items of the two scales and to what extent they assess the same construct. While it can be observed that there is a part of overlap between certain items, the primary difference between the two scales is in resolution: the CEQ focuses on lifetime experiences whereas the Greyson NDE scale focuses on one major event. Indeed, the latter focuses on the content of a specific type of experience (i.e., classical NDE or NDE-like) and its intensity at a phenomenological level, while the CEQ assesses a more broadly personality trait. Importantly, we further realized the correlation analysis without including the items showing an overlap with some items of the Greyson NDE scale and we still observed a significant positive correlation (see the Results section). This suggests that even in removing the critical items, we still observed an association between experiencers' engagement in fantasy and the reported intensity of the experience. Except the present study, no research has previously looked at the link between fantasy proneness and the occurrence of NDEs-like. The association may have important ramifications for studies addressing the phenomenon of NDEs-like and may open new theoretical perspectives.

To date, it is not clear whether NDEs are a randomly occurring phenomenon or whether some specific psychological factors play an important role in their generation (or recall). Although much has been learned regarding the NDE phenomenon *per se*, considerably less research has been directed at exploring the psychological mechanisms that might lead to the occurrence of such memories. Fantasy proneness (15) may constitute a psychological predisposition for the occurrence of NDEs-like. Because NDEs can subsequently induce life-changing consequences on the experiencers' set of values and attitudes toward death (22), further understanding of their cognitive functioning remains an important focus for clinical research.

## Ethics statement

This study was carried out in accordance with the recommendations of the ethics committee of the Faculty of Medicine of the University of Liège with written informed consent from all subjects. All subjects gave written informed consent in accordance with the Declaration of Helsinki. The protocol was approved by the ethics committee of the Faculty of Medicine of the University of Liège.

## Author contributions

CM and SL designed the protocol. CM, HC, and VC-V obtained the data. CM analyzed the data; CM, HC, HM, and SL: interpreted data. All authors contributed to the writing of the manuscript. CM and SL were the main investigators. All authors were involved in editing the paper and approved the final text.

### Conflict of interest statement

The authors declare that the research was conducted in the absence of any commercial or financial relationships that could be construed as a potential conflict of interest.
