# Supplementary material for: Fantasy Proneness Correlates With the Intensity of Near-Death Experience
Source: Front Psychiatry. 2018 Jun 7;9:190. doi: 10.3389/fpsyt.2018.00190 (PMC6001803; doi:10.3389/fpsyt.2018.00190)
Supplement: Supplementary file 1 [file Data_Sheet_1.docx]

Supplementary Material

**Fantasy proneness correlates with near-death experiences, but only when they developed outside a life-threatening context**

**Charlotte Martial^1*^, Héléna Cassol^1^, Vanessa Charland-Verville^1^, Harald Merckelbach^2^ & Steven Laureys^1^**

*** Correspondence:** Corresponding Author: cmartial@uliege.be

# Supplementary Table

| Table S1.  Percentage of TRUE responses for each CEQ item (each percentage calculated with respect to the total number of subjects in the subsample). | | | | | |
| --- | --- | --- | --- | --- | --- |
| CEQ items | NDErs | | Non-NDErs  non-LTS  *n*=20 | Controls | |
|  | LTS  *n*=51 | non-LTS  *n*=57 |  | LTS  *n*=50 | non-LTS  *n*=50 |
| *Item 1* | 6 | 12 | 15 | 18 | 12 |
| *Item 2* | 8 | 26 | 25 | 32 | 30 |
| *Item 3* | 20 | 18 | 5 | 8 | 12 |
| *Item 4* | 67 | 67 | 60 | 72 | 66 |
| *Item 5* | 25 | 44 | 35 | 28 | 30 |
| *Item 6* | 4 | 11 | 0 | 16 | 12 |
| *Item 7* | 47 | 56 | 40 | 22 | 26 |
| *Item 8* | 43 | 51 | 35 | 32 | 46 |
| *Item 9* | 14 | 23 | 10 | 10 | 6 |
| *Item 10* | 53 | 60 | 35 | 28 | 30 |
| *Item 11* | 37 | 51 | 35 | 26 | 32 |
| *Item 12* | 20 | 5 | 15 | 22 | 26 |
| *Item 13* | 10 | 14 | 10 | 8 | 8 |
| *Item 14* | 33 | 42 | 35 | 36 | 20 |
| *Item 15* | 10 | 14 | 25 | 16 | 14 |
| *Item 16* | 67 | 82 | 55 | 56 | 54 |
| *Item 17* | 29 | 40 | 30 | 18 | 8 |
| *Item 18* | 43 | 51 | 50 | 28 | 36 |
| *Item 19* | 27 | 32 | 35 | 20 | 20 |
| *Item 20* | 22 | 37 | 5 | 20 | 28 |
| *Item 21* | 57 | 65 | 20 | 22 | 18 |
| *Item 22* | 71 | 72 | 35 | 38 | 22 |
| *Item 23* | 51 | 81 | 45 | 14 | 8 |
| *Item 24* | 22 | 30 | 10 | 6 | 4 |
| *Item 25* | 27 | 46 | 0 | 6 | 0 |
| Notes: Data are percentages (%) ; NDErs=near-death experiencers ; LTS=life-threatening situation | | | | | |
